# Far-red light regulates phototactic behavior of benthic pennate epipelic diatoms under low irradiance

**DOI:** 10.1007/s11120-025-01195-w

**Published:** 2026-01-08

**Authors:** Jérôme Morelle, Alexandra Bastos, Luís F. Pereira, Silja Frankenbach, Johann Lavaud, João Serôdio

**Affiliations:** 1 CESAM – Centre for Environmental and Marine Studies and Department of Biology, University of Aveiro, Campus de Santiago, Aveiro, 3810-193 Portugal; 2 CIBIO, Centro de Investigação em Biodiversidade e Recursos Genéticos, InBIO Laboratório Associado, Universidade do Porto, Vairão, Portugal; 3 BIOPOLIS Program in Genomics, Biodiversity and Land Planning, CIBIO, Vairão, Portugal; 4 LEMAR - Laboratory of Marine Environmental Sciences, UMR 6539 CNRS, Univ Brest, Ifremer, IRD, Institut Universitaire Européen de la Mer, Technopôle Brest-Iroise, Plouzané, France

**Keywords:** Microphytobenthos, Vertical migration, Low light, Spectral sensing, Phototaxis

## Abstract

Diatom-dominated microphytobenthic communities are exposed to steep and dynamic light gradients in intertidal sediments. The vertical migration of epipelic pennate diatoms is a key adaptive trait enabling optimal light acquisition. However, it remains unclear whether these organisms can detect and respond to long-wavelength light, especially because deeper photic layers are enriched in far-red due to attenuation of shorter wavelengths. Here, we investigated the phototactic responses of a natural microphytobenthic biofilm, primarily composed of epipelic pennate diatoms, to long-wavelength light at two low irradiance levels (5 and 20 µmol photons m⁻² s⁻¹), using a custom-built multispectral LED illuminator. Red light (660 and 680 nm) induced a strong upward vertical migration and a high effective quantum yield of photosynthesis. Far-red light (720 and 740 nm) also triggered a significant upward migratory response, although more moderate than red light. In contrast, near-infrared wavelengths (770 and 810 nm) elicited no significant migratory activity, indistinguishable from the dark controls. Phototactic migration was observed even at 5 µmol photons m⁻² s⁻¹, suggesting a high sensitivity to light at intensities potentially below the photosynthetic compensation point. Our results provide evidence that benthic pennate diatoms can behaviorally respond to long-wavelength, low-intensity light. This response, likely mediated by phytochrome-like photoreceptors, suggests the existence of a low-light, long-wavelength sensing mechanism that enables diatoms to detect fine-scale spectral gradients as cues for surface detection and vertical positioning within the sediment matrix.

## Introduction

Intertidal sedimentary habitats are fundamental to marine coastal ecosystems, providing stability, shelter, and feeding grounds for a wide range of benthic organisms. Among these, microphytobenthos (MPB) biofilms, primarily composed of pennate diatoms, thrive on the sediment surface where they form dense and highly productive mats that can cover extensive areas (Hope et al. 2020). MPB plays a central role in primary production by forming the base of benthic food webs, contributing to nutrient cycling, sediment stabilization by binding particles together and reducing erosion, and supporting overall coastal ecosystem functioning (Underwood and Kromkamp 1999; Hope et al. 2020).

The growth and productivity of MPB are regulated by a variety of environmental factors (Underwood and Kromkamp 1999). In nutrient-rich systems, light availability at the sediment surface is a major driver of MPB activity (Brotas et al. 2003; Behrenfeld et al. 2004; Laviale et al. 2016). However, light availability in sedimentary habitats is highly variable, both spatially and temporally, with steep irradiance gradients across the sediment surface depending on grain size and the proportion of silt and sand (Kühl et al. 1994; Cartaxana et al. 2016; Morelle et al. 2020). In intertidal zones, these gradients are further modulated by diel light cycles and tidal immersion patterns (Coelho et al. 2011), as well as by shifts in the spectral quality of light penetrating the sediment (Kühl and Jørgensen 1994; Cartaxana et al. 2016). Moreover, the presence of MPB biofilms at the sediment surface also attenuate light penetration to deeper layers (Ploug et al. 1993; Morelle et al. 2018).

To cope with this variability in light conditions, most epipelic diatoms perform rhythmic vertical migrations synchronized with diel and tidal cycles (Serôdio 2021). This behavioral adaptation is believed to be a key factor in their evolutionary success, diversification, and high productivity in sedimentary environments. Vertical migration allows MPB to optimize light capture near the surface (De Brouwer and Stal 2001; Nakov et al. 2018; Poulsen et al. 2022; Morelle et al. 2024) and to access re-mineralized nutrients concentrated in deeper sediment layers (Orvain et al. 2003).

Due to the thin photic zone, typically < 1 mm (Kromkamp et al. 1998; Serôdio et al. 2023), a unique aspect of the sedimentary microenvironment is the spatial (vertical) proximity between direct exposure to high, full spectral light at the surface, and low, spectrally biased light below the surface, progressing to complete darkness. Notably, due to preferential absorption of photosynthetically active radiation (PAR) by overlying cells and pigments, deeper subsurface sediment layers tend to receive a light with a spectrum deviated towards the red/far-red light (Kühl and Jørgensen 1992; Cartaxana et al. 2016), as longer wavelengths penetrate deeper and are less scattered than shorter ones. In muddy fine sediments, these two extreme light conditions may be separated by only a few diatom cell lengths (Serôdio et al. 2001). Considering the frequent burial of cells below the photic zone due to sediment remixing, the ability to detect low-intensity, far-red light appears particularly advantageous, as it could function as a form of ‘surface sensing’. This capability may involve a phytochrome-based sensory system, as suggested by the presence of phytochrome genes in pennate diatoms (Depauw et al. 2012) and a phytochrome-mediated far-red signaling pathway in planktonic diatom species (Fortunato et al. 2016), and more recently, the diatom-phytochrome has been established as an optical depth detector that regulates photosynthetic acclimation across the underwater light spectrum (Duchêne et al. 2025). However, research on the motility responses of epipelic pennate diatoms to light has predominantly addressed ambient (actinic) irradiance levels (Prins et al. 2020), and evidence remains scarce regarding their ability to perceive low-intensity, spectrally shifted light (particularly in the red/far-red range) and the identity of the photoreceptors mediating such detection (Barnett et al. 2020).

Given the vertical migration behavior of epipelic pennate diatoms passively transported into deeper sediment layers, it seems ecologically significant that they have developed mechanisms to sense and respond to long-wavelength light under low light conditions. In this context, our study aimed to test the hypothesis that epipelic pennate diatoms are capable of detecting low-irradiance long-wavelength radiation (red, far-red, near-infrared) and respond through upward motility, focusing on levels below 20 µmol photons m⁻² s⁻¹, which represent 1% of an intertidal surface irradiance of 2000 µmol photons m⁻² s⁻¹ and thus to the lower limit of the euphotic zone under these conditions. To test this, we conducted a laboratory experiment using natural MPB diatom-dominated biofilms exposed to controlled spectral irradiance. Vertical migratory responses were quantified using imaging fluorescence technology, allowing non-invasive, high-resolution monitoring of biomass redistribution in response to the light treatments.

## Methods

### Sampling

In May 2025, samples of sediment colonized by diatom-dominated microphytobenthos were collected during a diurnal low tide on an intertidal mudflat of the Ria de Aveiro, Portugal (Vista Alegre; 40°35’00.9"N 8°41’11.5"W). Immediately after sampling, the sediment was homogenized and distributed into three separate black opaque 24-well plates. All wells were filled with 1.6 mL of homogenized sediment, measured using a 5 mL syringe (HENKE-JECT, Seoul, Republic of Korea). 200 µL of autoclaved seawater was added to each well to prevent desiccation, and the well plates were kept in the dark at room temperature (20 °C) until the beginning of the experiment, i.e., three hours before the diurnal low tide of the following day.

### Taxonomic identification

The remaining sampled sediment was used to collect cells using the lens tissue technique (Eaton and Moss 1966). The resulting cell assemblages were resuspended in 100 mL of filtered natural seawater, 2 mL was fixed using Lugol (5% v/v), and kept at 4 °C for future identification of the dominant species present. Identification was conducted under a Motic AE31 Inverted Microscope using 1 mL of the assemblage placed in a Sedgewick-Rafter chamber. Ten squares were analyzed within the chamber. All cells were counted and identified to the genus or species level. The mean percentage of each identified taxon was calculated from the counts across the 10 squares (*n* = 10).

### Experimental setup

**Fig. 1:**
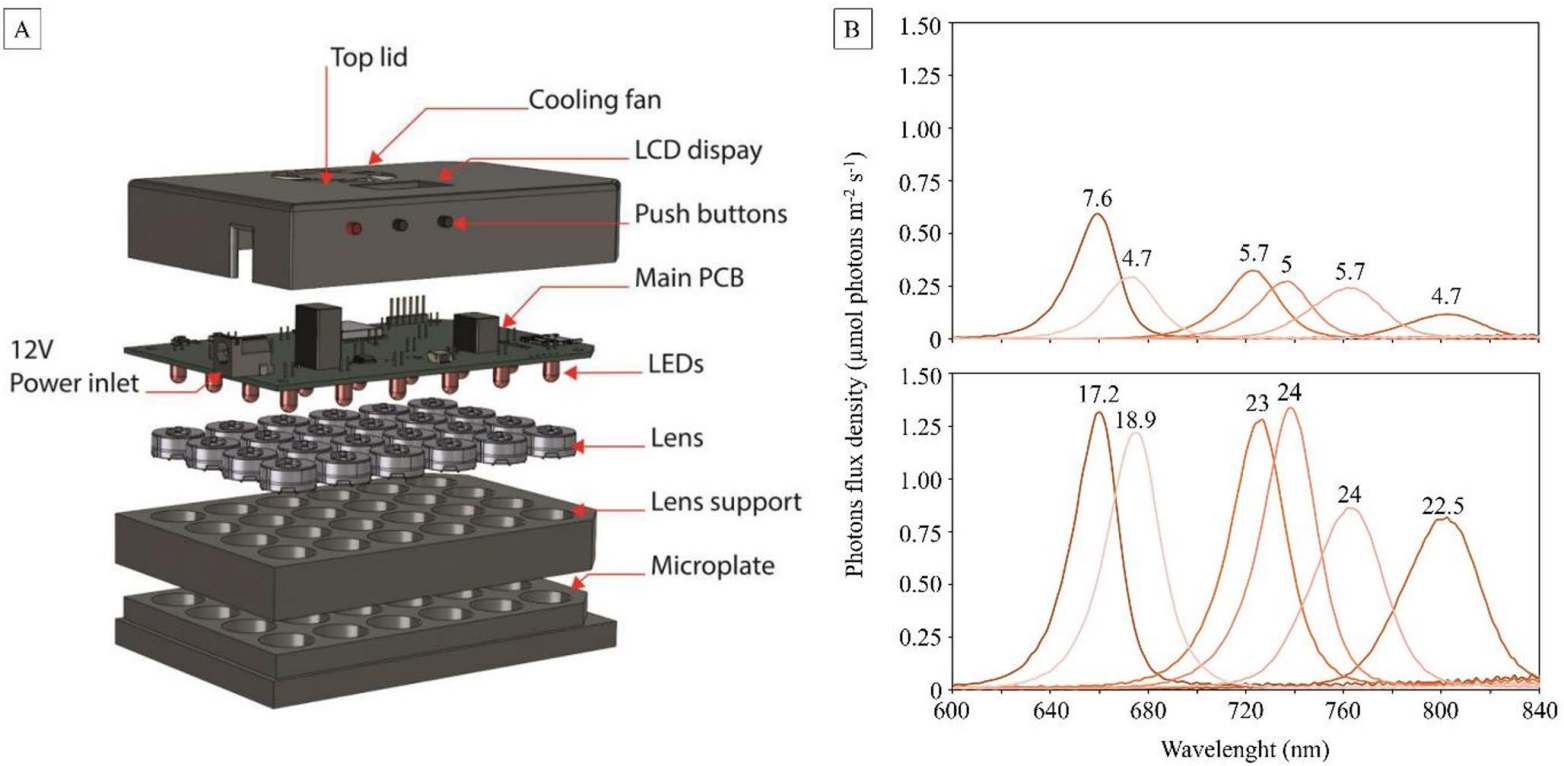
Multispectral LED illuminator used to expose replicated sediment samples to controlled light conditions. (**A**) Exploded view of the custom-built illuminator showing the spatial arrangement of the 24 individual LEDs, each aligned with a single well to independently deliver a specific wavelength and intensity (Pereira 2025). Peak emission wavelengths included red (660 and 680 nm), far-red (720 and 740 nm), and near-infrared (770 and 810 nm), with four replicates per wavelength per plate. (**B**) Emission spectra of the LEDs, showing the cumulative intensity values (µmol photons m⁻² s⁻¹) received at the surface of the sediment samples in the well plates. The top panel corresponds to the ~ 5 µmol photons m⁻² s⁻¹ experiment (range: 4.7–7.6), and the bottom panel to the ~ 20 µmol photons m⁻² s⁻¹ experiment (range: 17.2–24)

During the three hours preceding low tide, the plates were exposed to different light conditions. The first plate was kept in the dark for the entire duration of the experiment, serving as a dark control (*n* = 24 wells); the second and third plates were exposed to light intensities of about 5 and 20 µmol photons m⁻² s⁻¹, respectively. The individual wells, despite being exposed to identical light intensities, were exposed to different wavelengths, ranging from red visible light within the PAR spectrum (620–700 nm), to far-red light (700–750 nm), and to near-infrared light (NIR; 750–1400 nm). To achieve this, the well plates were placed under a custom-made multispectral illuminator (Pereira 2025) that allowed to independently expose each of the 24 wells to a single LED. The illuminators had LEDs with peak wavelengths in the red (660 and 680 nm), far-red (720 and 740 nm), and infra-red (770 and 810 nm) regions, with four replicates per wavelength (Fig. 1-A).

Both the intensity of light and peak of each LED applied in the different wells were measured (Fig. 1-B) using a calibrated spectroradiometer, quantum light meter SpectraPen mini (Photon Systems Instruments (PSI), Drásov, Czech Republic), positioned at the location corresponding to the sediment surface. Intensity of light was thus quantified as incident light expressed in µmol photon m^− 2^ s^− 1^. Although it would have been relevant to estimate pigment weighted light absorption (*Qphar*) as described in Prins et al. (2020), this measurement could not be performed with our experimental setup.

### Chlorophyll fluorescence measurements

Chlorophyll fluorescence was measured using a chlorophyll imaging fluorometer (Open FluorCam FC 800-O/1010, PSI, Drásov, Czech Republic), composed of two red LED panels (621 nm emission peak, 40 nm bandwidth; MLS13 × 13, PSI) providing the non-actinic measuring light (0.1 µmol m⁻² s⁻¹), and two red LED panels delivering saturating pulses (7500 µmol m⁻² s⁻¹; SL3500, PSI) (Serôdio et al. 2013). A user-defined protocol with a total duration of 2 s was used to measure the fluorescence parameters.

Initial dark-adapted fluorescence level (F_o_) was measured using only the non-actinic measuring light (< 0.1 µmol photons m⁻² s⁻¹) and without applying a saturating pulse, ensuring that the samples were not additionally stimulated by actinic light. This measurement was performed every 15 min on each of the three well plates used in the experiment. F_o_ values were used as a proxy for surface MPB biomass by monitoring changes in surface chlorophyll *a* fluorescence resulting from downward or upward migration (Serôdio et al. 2012; Laviale et al. 2016). An increase in F_o_ values was thus considered to reflect upward migration of microphytobenthic cells to the sediment surface, while a decrease in F_o_ values was considered indicative of downward migration of cells into the subsurface sediment layers (Perkins et al. 2010; Laviale et al. 2016; Barnett et al. 2020; Morelle et al. 2025).

After the three hours of exposure, at the time corresponding to the low tide, F_o_ measurements were followed by a high-intensity saturation pulse for 0.6 seconds to determine the relative maximum fluorescence level (F_m_’) (Alkimin et al. 2019). These fluorescence parameters were used to calculate the effective quantum yield of PSII (ΔF/F_m_’ = (F_m_’ − F)/F_m_’) (Genty et al. 1989).

### Data and statistical analyses

To account for variations in F_o_ values across replicates and treatments, these were expressed as percentages relative to the initial values recorded at the start of light exposure (T_0_ = 100%), serving as the baseline reference. To assess the significance of surface biomass (%) variation over time under the different treatment conditions (combinations of wavelength and light intensity), a Two-Way Repeated Measures ANOVA (Two-Factor Repetition) was performed using the SigmaPlot version 12.0. This analysis enabled the evaluation of the main effects (treatment and time) as well as their interaction. Additionally, a One-Way Analysis of Variance (ANOVA) was used to compare final values of the effective quantum yield of PSII (ΔF/F_m_’) among treatments.

## Results

### Community identification

The sampled microphytobenthic community was mainly dominated by diatoms with only a few Euglenoids, which accounted for 4.6 ± 3.9% of the total assemblage (Table 1). Among the diatoms, *Pleurosigma angulatum* was the most abundant species, representing 51.1 ± 7.6% of the counted individuals. This was followed by *Cylindrotheca colsterium* (17.8 ± 11.4%), *Gyrosigma balticum* and *G. wansbeckii* (9.2 ± 5.3%), and small diatoms primarily attributed to the genus *Navicula* sp. (6.0 ± 4.6%). *Diploneis* sp. and *Gyrosigma fasciola* accounted for 4.9 ± 3.3% and 2.6 ± 3.2% of the assemblage, respectively. The remainder of the community consisted of individuals of other pennate diatom taxa, each representing less than 1% of the total abundance.

**Table 1:** Taxonomic composition and relative abundance (mean ± SD) of the microphytobenthic community at the time of sampling. “Other pennate diatoms” includes taxa with individual contributions < 1%

Taxonomic Group / Species	Relative abundance (%)
*Pleurosigma angulatum*	51.1 ± 7.6
*Cylindrotheca closterium*	17.8 ± 11.4
*Gyrosigma balticum* and *G. wansbeckii*	9.2 ± 5.3
Small diatoms (*Navicula* sp.)	6.0 ± 4.6
*Diploneis* sp.	4.9 ± 3.3
*Gyrosigma fasciola*	2.6 ± 3.2
*Surirella gemma*	2.0 ± 2.3
Other pennate diatoms species (< 1% each)	1.8 ± 1.7
Euglenoids	4.6 ± 3.9

### Surface biomass

The Two-Way repeated measures ANOVA indicates that treatment, time, and their interaction were all highly significant (Treatment: F₁₂,₃₆ = 15.382, *p* < 0.001; Time: F₆,₁₈ = 14.989, *p* < 0.001; Treatment × Time: F₇₂,₂₁₆ = 23.727, *p* < 0.001).

**Fig. 2:**
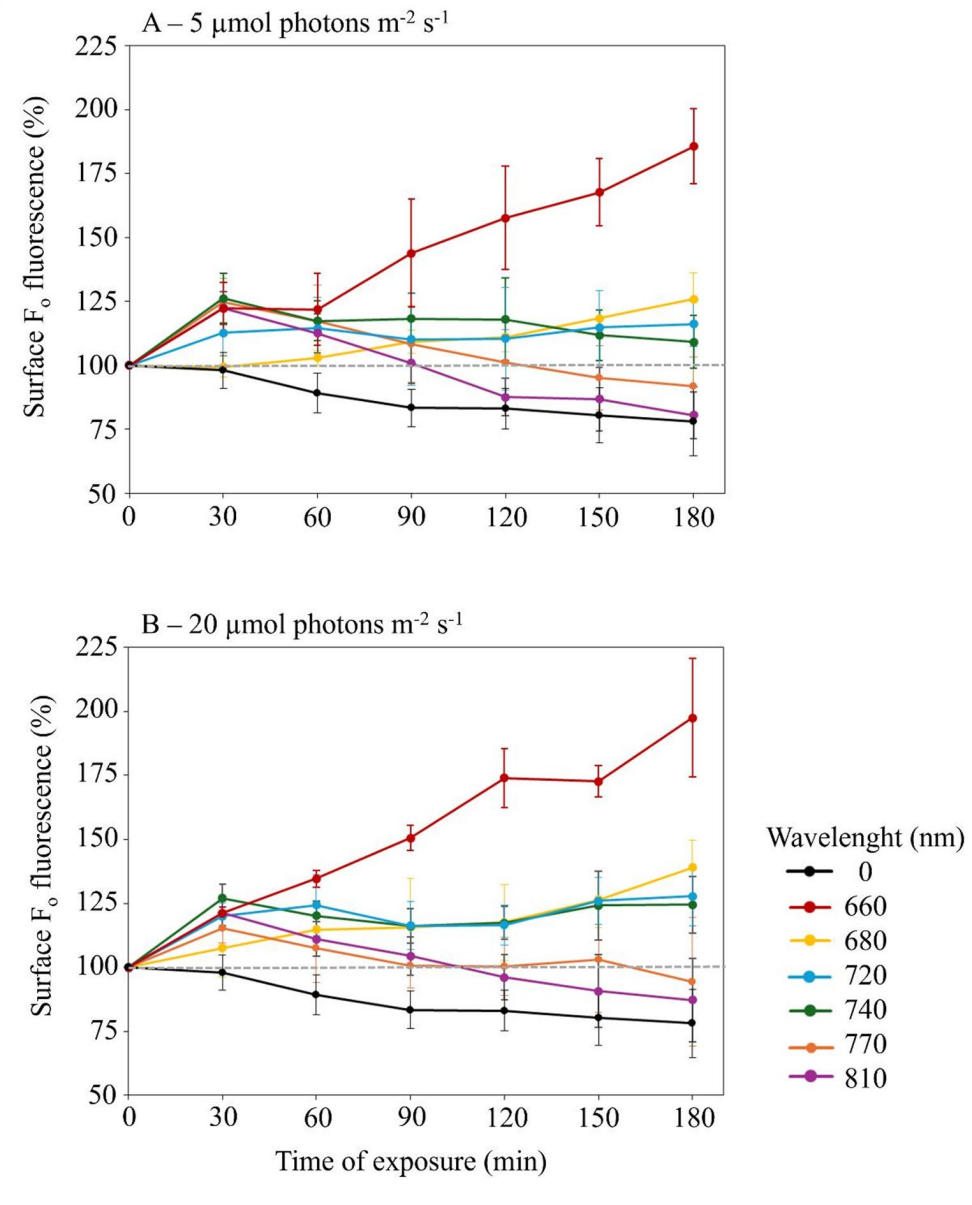
Spectral dependence of temporal changes in surface biomass under 5 µmol photons m⁻² s⁻¹ (**A**) and 20 µmol photons m⁻² s⁻¹ (**B**). Surface biomass values are expressed as a percentage relative to the values measured at the initial time point of exposure (T_0_ = 100%)

Under dark conditions, the surface biomass showed a significant decrease over time, reaching values at 90 and 180 min significantly lower than those at 0 and 30 min (*p* < 0.05), averaging − 22 ± 13% at 180 min relative to 0 min, indicating a gradual but significant loss of surface biomass in the absence of light (Fig. 2).

At an irradiance of about 5 µmol photons m⁻² s⁻¹, temporal changes in surface biomass varied significantly depending on light spectrum, reflecting wavelength-dependent redistribution of biofilm biomass and a gradient of behavioral responses across the spectrum (Fig. 2-A). In the red region, fluorescence increased significantly (*p* < 0.001) over time, reaching + 85.7% and + 25.9% on average at 180 min for 660 and 680 nm, respectively. Under far-red wavelengths, the surface biomass also increased although to a lesser extent. Under 720 nm, surface biomass increased by approximately + 5% at 180 min, while at 740 nm, an early increase in fluorescence at 30 min was followed by a plateau. In contrast, near-infrared (NIR) illumination led to a progressive decline in fluorescence, significantly decreasing by approximately − 8.3% and − 19.5% at 180 min for 770 nm and 810 nm, respectively.

At an irradiance of about 20 µmol photons m⁻² s⁻¹, the changes in surface biomass showed similar wavelength-dependent behaviors with slightly higher but non-significant effects when compared to 5 µmol photons m⁻² s⁻¹ (Fig. 2-B). In the red range (600 and 680 nm), fluorescence increased significantly over time, reaching average peaks of + 97.6% at 660 nm and + 38.9% at 680 nm, after 180 min (*p* < 0.001). Far-red light (720 and 740 nm) induced a transient increase in fluorescence that plateaued thereafter. Conversely, NIR wavelengths (770 and 810 nm) consistently led to a significant decline in fluorescence, dropping by approximately − 6% and − 13% at 180 min (*p* < 0.001), not significantly different from the dark control.

**Fig. 3:**
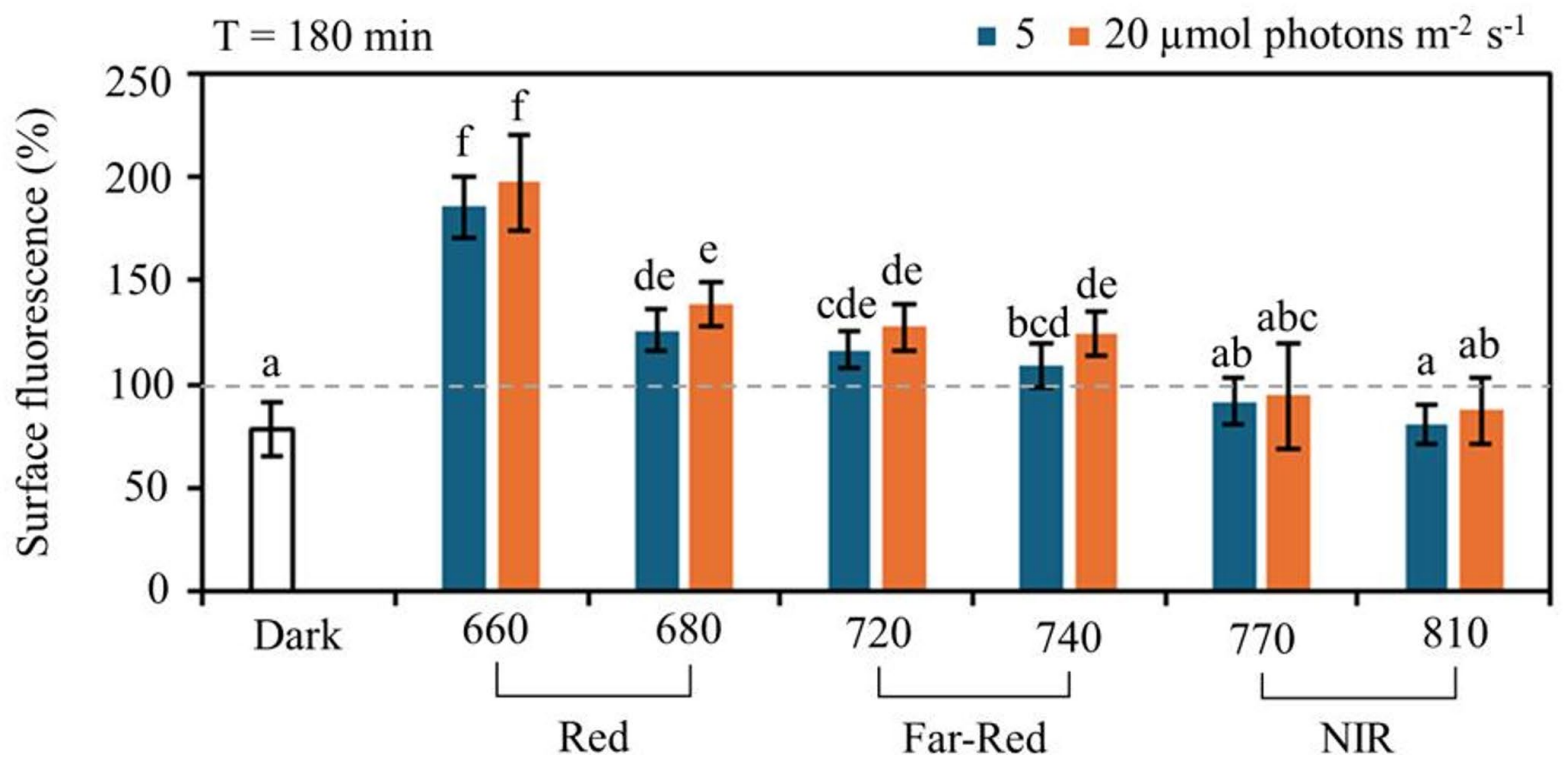
Surface biomass (%) measured after 180 min of exposure to different light intensities and wavelengths. Samples were exposed to red (660 and 680 nm), far-red (720 and 740 nm), and near-infrared (770 and 810 nm) light at 5 and (blue bars) and 20 µmol photons m⁻² s⁻¹ (orange bars). The control (Dark) corresponds to samples kept in darkness. Values represent mean ± SD (*n* = 24 for control and *n* = 4 for 5 and 20 µmol photons m^− 2^ s^− 1^). Different letters indicate statistically significant differences between treatments based on post-hoc test after a 2-way repeated measures ANOVA (*p* < 0.05)

At the end of exposure (T = 180 min), the magnitude of the changes in surface biomass across all spectral treatments followed a consistent pattern, revealing a wavelength-dependent response gradient (Fig. 3). Under both irradiance levels, the net effect on surface biomass (compared to the dark control) was ordered as follows: 660 nm > 680 > 720 > 740 > 770 > 810 > 0 (dark control). The biomass response pattern cannot be explained by the small variations in irradiance across LEDs (Fig. 1), suggesting that spectral quality, rather than total PAR, is the primary driver of surface biomass variation in this experiment. This ranking highlights a progressive decrease in biomass from red wavelengths to dark conditions, with longer wavelengths inducing significantly lower biomass accumulation. The differences were particularly pronounced at 660 nm, where increases in biomass were significantly higher than at all other wavelengths, emphasizing the enhanced biofilm response under PAR red light. At 680 nm and in the far-red region (720 and 740 nm), biomass accumulation was lower than at 660 nm but remained significantly higher than under the dark control. In contrast, near-infrared wavelengths (770 and 810 nm) did not induce any significant difference compared to the dark control. Despite the lack of statistical significance, slightly higher values were consistently recorded at 20 than 5 µmol photons m⁻² s⁻¹ for each wavelength.

### Effective quantum yield of PSII (ΔF/F_m_’)

**Fig. 4:**
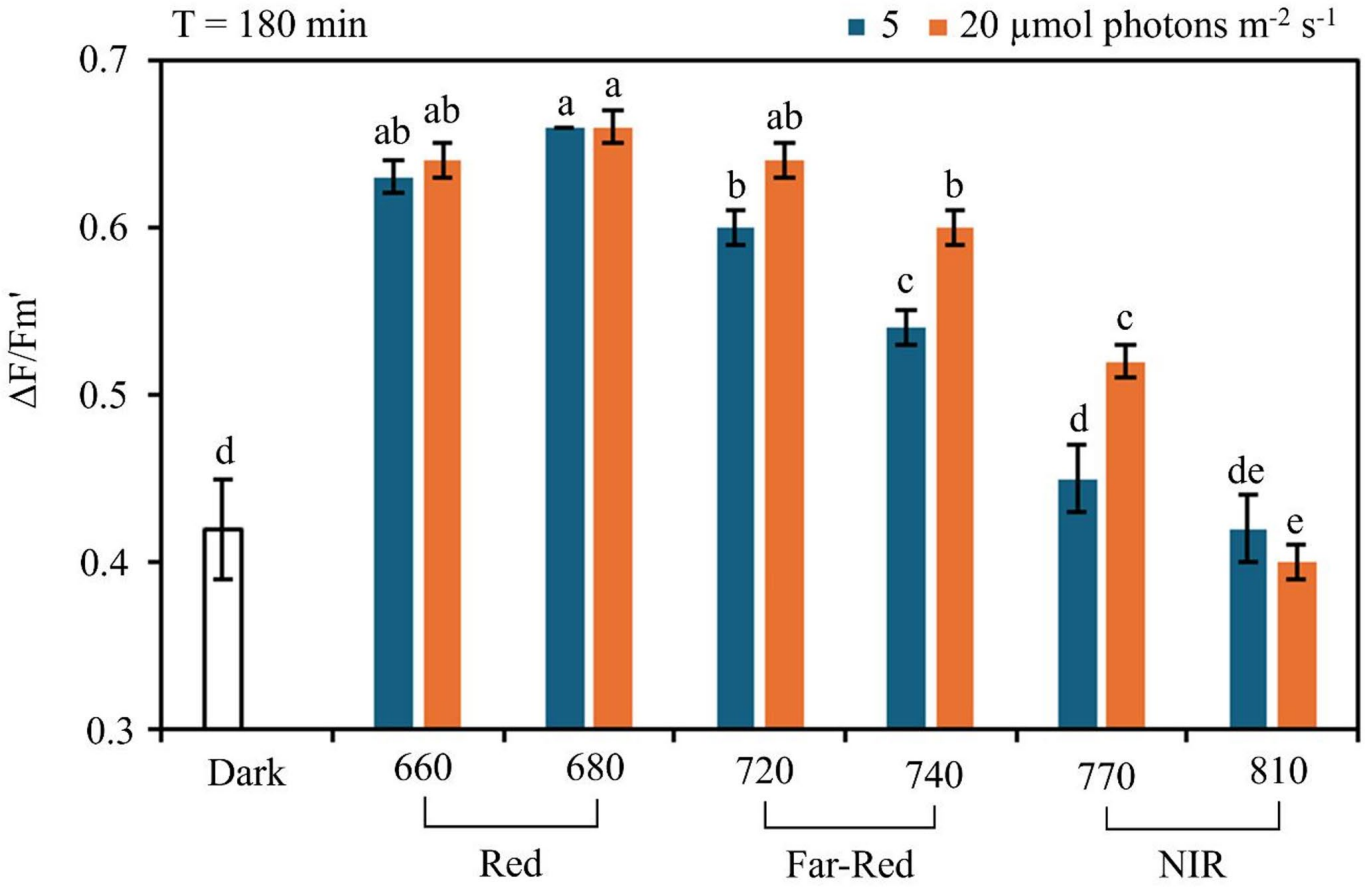
Effective quantum yield of PSII (ΔF/Fm’) measured after 180 minutes of exposure to different light intensities and wavelengths. Samples were exposed to red (660 and 680 nm), far-red (720 and 740 nm), and near-infrared (770 and 810 nm) light at 5 and (blue boxes) and 20 µmol photons m⁻² s⁻¹ (orange boxes). The control (Dark) corresponds to samples kept in darkness. Each box represents the distribution of ΔF/F_m_’ values (*n* = 4 for samples, *n* = 24 for Dark). Different letters indicate statistically significant differences between treatments based on post-hoc test after a 1-way ANOVA (*p* < 0.05)

The effective quantum yield of PSII (ΔF/F_m_’), measured at the end of exposure (T = 180 min), also varied significantly with the spectral quality of the light (p < 0.001; Fig. 4). In the dark control, the mean ΔF/F_m_’ was 0.42 ± 0.03 (*n* = 24). Under red light (660 and 680 nm), values were significantly higher, averaging 0.63 ± 0.01 and 0.66 ± 0.01, respectively. Under far-red light (720 and 740 nm), ΔF/F_m_’ values were intermediate. At 720 nm, the average value was 0.62 ± 0.02 across both intensities. At 740 nm, values differed significantly between intensities with 0.54 ± 0.01 at 5 µmol photons m⁻² s⁻¹ and 0.60 ± 0.01 at 20 µmol photons m⁻² s⁻¹. In contrast, near-infrared light (770 and 810 nm) resulted in significantly lower ΔF/F_m_’ values. At 770 nm, the mean ΔF/F_m_’ was 0.45 ± 0.02 under 5 µmol photons m⁻² s⁻¹, not significantly different from the dark control, and 0.52 ± 0.01 under 20 µmol photons m⁻² s⁻¹. At 810 nm, the mean ΔF/F_m_’ was 0.41 ± 0.02 across both intensities, and not significantly different from the dark control at 5 µmol m⁻² s⁻¹ (Fig. 4).

## Discussion

### Sensitivity to low irradiance levels

The present study aimed to fill a key knowledge gap in MPB photobiology by investigating the ecological significance and behavioral consequences of long-wavelength light detection under low irradiance conditions, representative of natural sub-surface light environments in intertidal sediments. The observed positive phototactic response of epipelic pennate diatoms at 660 nm under irradiances as low as 5 µmol photons m⁻² s⁻¹ highlights their sensitive photosensing capacity. This sensitivity is ecologically meaningful given the strong attenuation of light within intertidal sediments (Kühl et al. 1994; Kromkamp et al. 1998; Cartaxana et al. 2016; Frankenbach et al. 2020). Morelle et al. (2018) measured light attenuation coefficients (k_d_) ranging from 1 mm⁻¹ in sandy sediments to 4 mm⁻¹ in muddy sediments, consistent with previous estimates for dry sand and diatom-colonized sediments (Kühl and Jørgensen 1994). Assuming a maximum surface irradiance of ~ 2000 µmol photons m⁻² s⁻¹, similar to midday solar levels in intertidal zones, irradiance would fall to 5 µmol photons m⁻² s⁻¹ within 1.5 to 6 mm of sediment depth. This range aligns well with the known vertical distribution of epipelic diatoms in both sandy and muddy sediments (MacIntyre and Cullen 1995; Morelle et al. 2020). The observation that diatoms initiated upward migration under 5 µmol photons m⁻² s⁻¹ applied at the sediment surface, and considering the rapid attenuation of light within sediment, suggests that the cells triggering this response were exposed to even lower irradiance, potentially in the sub-micromolar photon range. This indicates that the threshold for light detection and behavioral response in epipelic diatoms lies below typical compensation points, where gross O_2_ production balances respiratory consumption (Hoffmann et al. 2019; Prelle et al. 2022). Moreover, because the amount of light actually absorbed by the cells (*Qphar*) is likely lower than the incident light measured at the sediment surface (Prins et al. 2020), the effective photon absorption triggering this response was likely even lower than our estimates. Although *Qphar* could not be quantified here, this concept reinforces the idea that epipelic diatoms respond to very small amounts of absorbed light. However, as we cannot exclude the involvement of additional cues such as chemical communication (Venuleo et al. 2017), our findings demonstrate that a surface irradiance of 5 µmol photons m⁻² s⁻¹ is sufficient to trigger upward migration. Furthermore, the slightly enhanced migratory response observed at 20 µmol photons m⁻² s⁻¹ suggests a sensitive dose-dependent relationship between irradiance and surface biomass accumulation. This indicates that photokinesis in epipelic pennate diatoms operates effectively across fine-scale light gradients, allowing precise and dynamic vertical positioning within the sediment (McLachlan et al. 2009; Morelle et al. 2024).

In our study, epipelic diatoms in complete darkness migrated downward within the sediment, resulting in a measurable decrease in surface chlorophyll fluorescence. While this downward migration contrasts with the upward movement based on endogenous rhythms during the three hours preceding low tide described by some studies (Consalvey et al. 2004a; Serôdio 2021), observations similar to ours have been reported. Coelho et al. (2011) showed that upward migration of intertidal benthic microalgae depends on both endogenous rhythms and light exposure (exogenous component), and that the alignment between the internal rhythm and the day-night cycle critically affects the magnitude of the endogenous control of vertical migration. In our dark control, the endogenous component may not have been sufficient to induce or maintain cell accumulation at the surface, and biofilms may have begun to disassemble as cells move downward (Coelho et al. 2011). Nevertheless, the dark treatment serves as a true control by definition, representing the absence of the light treatment under study. Considering that the diatoms were in healthy condition and that samples were identical across all treatments, the lack of upward migration in darkness, compared with the response under very low light, further supports our conclusion that epipelic diatoms are highly sensitive to light and can respond to extremely low irradiance levels, rather than relying solely on endogenous or physiological effects.

This ability to detect and respond to low irradiance levels highlights the efficiency of the photoreceptive system in epipelic pennate diatoms and reinforces the ecological relevance of phototactic migration as an adaptive trait. It supports the idea that photoinduced migration could be a highly adapted response to the steep and dynamic light gradients characteristic of intertidal sediments, likely contributing to the ecological success of these diatoms in such highly variable environments.

### Migratory response to long-wavelength light

Beyond light intensity, the spectral quality of the light stimulus plays a critical role in shaping the phototactic behavior of epipelic pennate diatoms (McLachlan et al. 2009; Prins et al. 2020). Our results reveal a clear hierarchy in migratory responses depending on long-wavelength light. While red light (660–680 nm), corresponding to the red range of PAR, elicited a strong upward vertical migration, exposure to far-red wavelengths (720–740 nm) induced a moderate yet significant upward migratory response.

Far-red light has previously been reported to elicit contrasting responses in benthic diatoms, including stimulation of downward migration and photophysiological effects on chlorophyll fluorescence, notably a reduction in F₀ (Consalvey et al. 2004b). In this context, the increase in surface fluorescence observed here under far-red illumination compared with darkness indicates that, at the community level, surface biomass was maintained or enhanced. If far-red exposure reduces F₀ at the level of individual cells, as previously suggested, this would further reinforce the interpretation that far-red light promotes surface biomass accumulation in our study, despite potential photophysiological effects. This suggests that far-red light could elicit context-dependent migratory and photophysiological responses in benthic diatoms.

In line with this interpretation, our results suggest the involvement of specialized photoreceptors, such as phytochromes, capable of detecting far-red light as an environmental cue (Fortunato et al. 2016). Far-red–induced migration occurred despite far-red light lying outside the conventional PAR range (400–700 nm), suggesting that this response may rely primarily on light perception rather than energy conversion. However, the relatively high ΔF/F_m_’ values observed under 720 nm, which were not statistically different from those under red light, leave open the possibility of some residual photosynthetic activity under this specific far-red radiation.

Together, these observations suggest a gradient of functional roles, with red light supporting both photosensory and energetic processes, whereas far-red light, less efficient for photosynthesis, may function primarily as a spectral signal.

This highlights the complexity of light sensing in diatoms, which involves both photoreceptors and photosynthetic pigments, each activating regulatory pathways. While photoreceptors can initiate specific signaling cascades upon light detection (Duanmu et al. 2017), photosynthetic pigments absorb a broader portion of the light spectrum and may regulate responses through retrograde signaling from the chloroplast (Jaubert et al. 2022; Morelle et al. 2025). These parallel pathways may converge to modulate cellular behaviors such as vertical migration.

Far-red perception, likely mediated by phytochrome-like photoreceptors, could act upstream of such cascades to modulate physiological processes beyond photosynthesis. It was shown that photoreceptors in microalgae can be coupled with photo-activated proteins responsible for a variety of physiological functions (Sushmita et al. 2020). As gliding motility relies on metabolically demanding processes such as exopolysaccharide secretion and cytoskeletal rearrangement (Poulsen et al. 1999; Tolhurst et al. 2003). This raises the possibility that light perception in the far-red region could regulate metabolic processes linked to vertical motility through such photoactivated proteins, directly linking environmental light cues to the migratory behavior of benthic epipelic diatoms. This hypothesis on the implication of the phytochrome in the vertical migration of microphytobenthic diatoms is supported by recent genomic analyses that revealed an expansion of the phytochrome family in some biofilm-forming diatoms, suggesting that the diatom phytochrome genes may represent an adaptation to their specific ecological niches (Osuna-Cruz et al. 2020).

In contrast, near-infrared wavelengths (770–810 nm) fail to trigger a migratory response, yielding surface biomass changes and ΔF/F _m_’ values similar to those observed in darkness, indicating either an absence of detection or the interpretation of the signal as unfavorable.

In parallel, the hypothesis that vertical migration under long-wavelength light could be driven or modulated by photosynthetic activity cannot be excluded. As previously suggested by Wenderoth and Rhiel (2004), the absorption of red light (680 nm) by photosynthetic pigments such as chlorophylls and carotenoids may indirectly trigger migration. This would represent a photosynthesis-driven response, rather than mediated by specific photoreceptors. Moreover, far-red LED spectra are not perfectly monochromatic but have a finite bandwidth overlapping with shorter red wavelengths, meaning that a small fraction of photons could still be absorbed by photosynthetic pigments. However, several lines of evidence suggest that photosynthesis alone cannot explain the responses observed here: far-red light (720–740 nm) lies largely outside the optimal absorption range for photosynthesis and the very low irradiances applied are below typical compensation points for oxygenic photosynthesis (Hoffmann et al. 2019; Prelle et al. 2022).

Furthermore, recent studies have reported the induction of red-shifted antenna complexes under prolonged exposure to red or low-light conditions in some pennate diatoms (Fujita and Ohki 2004; Herbstová et al. 2017; Wang et al. 2023). Such pigment-protein reorganizations could, in principle, modify light absorption and energy transfer properties, potentially influencing cell behavior. However, these adaptive changes typically require several days to develop under sustained, red-biased illumination. Given that our experiments lasted only three hours, it is unlikely that such long-term chromatic acclimation occurred, and we therefore consider its contribution to the observed responses to be minimal.

Overall, our results indicate that both red and far-red light influence the vertical migration and surface accumulation of benthic epipelic diatoms, likely through a combination of mechanisms involving photoreceptor-mediated signaling, possibly through phytochrome-like photoreceptors, and photosynthetically induced migration via pigment absorption. Further experiments will be needed to disentangle the respective contributions of these processes and better understand their interplay.

### Ecological implications

This ability of epipelic pennate diatoms to respond both to low irradiance levels and to specific long-wavelength spectral ranges has significant ecological implications for microphytobenthic sediment colonization and primary production. It is well established that red and far-red light undergo different patterns of attenuation, diffusion, and absorption in the sediment matrix, at fine spatial scales (Kühl and Jørgensen 1994). Our results demonstrate that benthic epipelic diatoms are capable of detecting light of both wavelength regions, suggesting that they could use the red to far-red ratio (R: FR; I₆₆₀/I₇₃₀) as a proxy for vertical position or light availability within the sediment matrix. Because red and far-red photons interact differently with interstitial water, organic matter, and mineral particles, the R: FR ratio naturally shifts with depth, creating a predictable spectral gradient. Data from a previous study conducted in a Tagus estuary mudflat (Cartaxana et al. 2016) showed that the R: FR ratio decreases by approximately 1.5 orders of magnitude within the first 100 μm of sediment depth, and by nearly 3 orders of magnitude beyond 700 μm, revealing a steep fine-scale gradient particularly relevant given that some epipelic diatoms can reach sizes exceeding 200 μm length (Bastos et al. 2025).

This mechanism mirrors well-known responses in terrestrial plants, where a low R: FR ratio, characteristic of shaded environments, triggers adaptive responses collectively known as the shade-avoidance syndrome (Smith 1982; Ballaré et al. 1990). By analogy, it is plausible that diatoms similarly exploit this spectral ratio gradient as an environmental cue to assess their vertical position in the sediment and to initiate appropriate migratory behavior, particularly under low-light conditions. In this context, the ability to discriminate between red and far-red light may provide a finely tuned mechanism to guide vertical phototaxis in complex sediment environments.

Altogether, such multispectral sensing may enable diatoms to couple their vertical motility with a fine-scale interpretation of the ambient light spectrum, supporting an advanced adaptive strategy for optimizing light exposure to variable sedimentary habitats. Moreover, phytochromes were shown to exhibit distinct absorption or response spectra in different diatom species (Fortunato et al. 2016), which could contribute to species-specific differences in light perception, the sensitivity to red and far-red wavelengths, and different migration dynamics (Underwood et al. 2005).

The observed responsiveness to red and far-red light also suggests that diatoms may use spectral cues not only to locate favorable photic microenvironments but also to anticipate temporal changes in light conditions, such as those induced by tidal cycles or cloud cover. During tidal emersion, as the overlying water is flushed out, red and far-red wavelengths progressively reappear at the sediment surface, whereas they were previously strongly attenuated by water during high tide. Sensitivity to red and far-red light, typically associated with phytochrome-like photoreceptors, may thus offer both spatial and temporal advantages for benthic epipelic diatoms, by serving as an early indicator of emersion. This ability could enable them to initiate upward migration in anticipation of increasing light availability, thereby conferring a competitive advantage during the short photic windows.

Overall, the high photosensory precision displayed by epipelic diatoms emphasizes the role of light not only as an energy source but also as an environmental signal that governs spatial behavior. These findings highlight the evolutionary advantages conferred by such sensitivity in fluctuating coastal environments and underscore the need to further explore the molecular basis of light perception in benthic microalgae.

### Perspectives

While our results provide strong evidence for the involvement of red and far-red light in regulating vertical migration of benthic epipelic diatoms, several key questions remain to be addressed. Confirming the presence and functional role of phytochrome-like photoreceptors through gene expression analyses profiling and functional genetics (e.g., knock-out or knock-down mutants) is essential to establish causal links (Duchêne et al. 2025). Additionally, testing the effects of varying red-to-far-red (R: FR) light ratios on migratory behavior will clarify the functional significance of this spectral cue in light sensing and behavioral response of benthic epipelic diatoms. Moreover, now that the involvement of red and far-red light in phototactic responses of natural microphytobenthic biofilms is confirmed, exploring interspecific variability in sensitivity to long-wavelength light is important, as different diatom species may exhibit distinct sensitivities and migratory strategies (Jesus et al. 2023). Overall, this study provides new insights into the mechanisms linking light sensing to motility in benthic epipelic diatoms, an area that remains largely unexplored, identifying promising avenues for future mechanistic and ecological research.

## Data Availability

The data that support the findings of this study are available from the corresponding author upon reasonable request.
